# Relation between Proepileptic Activity of Indomethacin and AdrenalGland Hormones

**Published:** 2012

**Authors:** Ahmet Hacimuftuoglu, Halis Suleyman, Elif Cadirci, Abdulmecit Albayrak, Beyzagul Polat, Hamit Hakan Alp, Zekai Halici

**Affiliations:** a *Department of Pharmacology, Faculty of Medicine, Ataturk University, 25240, Erzurum, Turkey.*; b *Department of Biochemistry, Faculty of Medicine, Ataturk University, 25240, Erzurum, Turkey.*

**Keywords:** Epilepsy, Indomethacin, Adrenaline, Corticosterone, Rat

## Abstract

The role of inflammation has been shown in the pathogenesis of epilepsy, while glucocorticoids and adrenaline have anti-inflammatory effects. The aim of the present study was to investigate the effects of adrenaline, prednisolone, and indomethacin on caffeine-induced epilepsy (epileptiform activity) in rats and to examine the mechanism of the pro-epileptic effect of indomethacin. The adrenalectomized rats that had been given only adrenaline (the control group) did not die; however, adrenaline did not prevent the adrenalectomized rats which were given prazosin, phenoxybenzamine, yohimbine, metoprolol, and propranolol from dying. In the rats given propranolol + adrenaline, the rate of death was 100%, while this rate was 50% in the groups receiving prazosin + adrenaline, phenoxybenzamine + adrenaline, and metoprolol + adrenaline. The rate was 75% in the group given yohimbine + adrenaline. Prednisolone increased the degree of convulsion in adrenalectomized rats. Over-reduction in the blood catecholamine level made epileptogenesis more severe. It was observed that adrenaline pressed epileptogenesis via its own receptors (*α *- 1, *α *- 2, *β *- 1, *β *- 2). It was also revealed that all of the adrenergic receptors were responsible due to antiepileptic activity; *β *- 2 receptors played the most important role. It was observed that both acute and chronic indomethacin administration reduced the catecholamine levels. The situation in which acute administration of indomethacin did not affect epileptogenesis might originate from the fact that the structure of indomethacin did not significantly increase the corticosterone level.

## Introduction

Epilepsy is a reaction of the brain that has a focal or generalized characteristic. It has been shown in the literature that epilepsy has a connection with brain trauma, brain abscess, and fever diseases (1). Many studies have shown the role of inflammation in the pathogenesis of epilepsy. In rats, inflammation has been observed in the regions where epilepsy occurs and expands; the same situation has been observed in the human hippocampal region as well (2). An increase in the number of pro-inflammatory cytokine receptors such as IL-1 and IL-2 has been shown in the brain tissue of rats with epileptic temporal lobes. This increase has demonstrated a parallel to the degree of inflammation (3). In generalized seizures, the role of cytokines such as IL-1 and TNF-*α *has been observed in rats as well, and it has been proved that inflammation has an active role in the pathophysiology of these seizures (4). Vezzani *et al. *presented the degree of inflammation in epilepsy in their clinical and experimental investigations (5). The amounts of pro-inflammatory cytokines (IL-1, IL-1*β*, TNF-*α*, IL-6) in healthy brains are very small; but in epileptic models, it was observed that the amounts of these cytokines rapidly increased (6, 7). Administration of kainic acid caused overstimulation of glutamate receptors and was linked to overstimulation inflammatory excitotoxicity (8). An increase in COX-2 activity is an important cause of neuroinflammation (9). Furthermore, an increase in COX-2 activity caused damage in the brain (10). However, indomethacin, which has a potent anti-inflammatory effect by inhibiting COX enzymes, is contraindicated in epilepsy (11). It is well known that glucocorticoids (cortisol) cause an anti-inflammatory effect via the phospholipase A-2 inhibitive effect (12). But it has been shown that in low doses glucocorticoid does not change the degree of epilepsy, and in high doses, increases this degree (13).

An adrenal gland hormone, adrenaline has a potent anti-inflammatory effect via stimulation of the *β*-2 adrenergic receptors (14). It was reported that the *β *adrenergic system has potent antiepileptic activity (15). In the literature, the antiepileptic activity of noradrenaline and dopamine was mentioned, but no reports about the antiepileptic activity of adrenaline were found. In addition, the pro-epileptic effect of indomethacin has not been clarified.

Therefore, the aim of the present study was to investigate the effects of adrenaline, prednisolone, and indomethacin on caffeine-induced epilepsy (epileptiform activity) in rats and to examine the mechanism of the pro-epileptic effect of indomethacin.

## Experimental

*Animals*

A total of 162 male Albino Wistar rats weighing 220–230 g were obtained from the Ataturk University Medicinal and Experimental Application and Research Center for use in this study. The animals were divided into treatment groups before the experimental procedures were initiated. The animals were housed and fed under standard conditions in a laboratory where the temperature was kept at 22°C. Animal experiments were performed in accordance with the national guidelines for the use and care of laboratory animals and were approved by the local animal care committee of Ataturk University.

*Chemicals*

Adrenaline was purchased from Drogsan (Turkey); prednisolone from Fako (Turkey); propranolol from Sanofi Synthelabo (Turkey); prazosin from Pfizer (Turkey); and metoprolol from AstraZeneca (Turkey). Yohimbine, phenoxybenzamine, and caffeine were obtained from Sigma Co., and thiopental sodium indomethacin was obtained from Deva Holding (İstanbul, Turkey).

*Caffeine-induced epileptiform activity in intact and adrenalectomized rats*

In this part of the study, 300 mg/kg of caffeine (16) was injected intraperitoneally in the intact and adrenalectomized rat groups . After injection, the animals were embedded into a glass box immediately, and observation was started. The time (latent period) was assessed by chronometer until tonic-clonic convulsions started. The number of convulsing animals and dead animals within two hours was determined at the end of the observation. Anticonvulsant (anti-epileptiform) activity was evaluated by comparing the results of the intact rats and the adrenalectomized rats (17).

*Caffeine-induced epileptiform activity in the adrenalectomized rats given adrenaline and prednisolone*

In this experiment, one group of the adrenalectomized rats was injected with 100 mg/Kg of adrenaline (18), and the other group was injected with 5 mg/Kg of prednisolone (19) intraperitoneally. Ten minutes after the injection, 300 mg/Kg of caffeine was injected intraperitoneally in these two rat groups. The anticonvulsant activities of the drugs were assessed as mentioned above.

*Effect of adrenaline on caffeine-induced epileptiform activity in the adrenalectomized rats given prazosin, phenoxybenzamine, yohimbine, metoprolol, and propranolol*

In this part of the xperiment, prazosin (5 mg/Kg, per oral), phenoxybenzamine (20 mg/Kg, intraperitoneal), yohimbine (10 mg/Kg, intraperitoneal), metoprolol (50 mg/Kg, per oral), and propranolol (40 mg/Kg, per oral) were applied to the adrenalectomized rats. Thirty minutes after the administration of phenoxybenzamine and yohimbine and one hour after the administration of prazosin, metoprolol, and propranolol, 100 mg/Kg of adrenaline was injected intraperitoneal in all the rat groups. Ten minutes after the adrenaline injection, 300 mg/Kg of caffeine was injected intraperitoneal in all groups. The anticonvulsant effect was assessed as mentioned above.

*Effects of acute and chronic indomethacin administration on caffeine-induced epilepsy in rats*

In this section, 10 mg/Kg of indomethacin (20) was administrated orally to both the intact and adrenalectomized rats. For acute administration, three hours after the single-dose indomethacin intake, 300 mg/kg of caffeine was injected intraperitoneal into the intact and adrenalectomized rats. The epileptiform activity of the intact and adrenalectomized rats was evaluated versus the control group as mentioned above. For chronic administration, 10 mg/Kg of indomethacin was given for 7 days per oral to the rats. Three hours after the last dose, 300 mg/Kg caffeine was injected intraperitoneal into the intact and adrenalectomized rats. The other procedures were carried out as acute administration.

*Biochemical analysis*

*Acute and chronic administration of indomethacin*

In parallel to epileptiform activity tests, 10 mg/Kg of indomethacin single dose was administrated orally to rats. For chronic administration, 10 mg/Kg of indomethacin was given for 7 days per oral to the rats. Both groups of intact and adrenalectomized rats that had been given acute and chronic indomethacin, underwent high-dose thiopental anesthesia. The rats’ blood samples were collected from their hearts in 2 mL EDTA vacuum tubes. Then, the tubes were sent to a biochemical laboratory to determine the adrenaline, noradrenaline, dopamine, and corticosterone levels.

*Measurement of adrenaline, noradrenaline, and dopamine levels in rats*

Blood samples were collected from the hearts of rats in 2 mL EDTA vacuum tubes to determine the adrenaline, noradrenaline, and dopamine levels. Within 15 min of venesection, the EDTA samples for the adrenaline, noradrenaline, and dopamine measurements were placed on ice and centrifuged at 3500 g for 5 min. After centrifugation, the plasma adrenaline, noradrenaline, and dopamine concentrations were measured by an isocratic system using a high-performance liquid chromatography (HPLC) pump (model Hewlett Packard Agilent 1100) (flow rate: 1 mL/min; injection volume: 40 μL; analytical run time: 20 min) and an electrochemical detector. We used a reagent kit for HPLC analysis of the catechol-amines in the plasma serum (Chromsystems, Munich, Germany).

*Measurement of corticosterone levels in rats*

Blood samples were collected from the hearts of rats in 2 mL EDTA vacuum tubes to determine the corticosterone levels in rats. Samples were centrifuged at 3500 g for 10 min. The samples for the measurement were frozen and kept at -80°C until measurement day. The plasma was separated and extracted with 5 mL of ethyl acetate (betamethasone as the internal standard), and then the extract was washed with sodium hydroxide (0.1 M) and water. After evaporation of the ethyl acetate, the residue was dissolved in the mobile phase (acetonitrile-water-acetic acid-TEA, 22:78:0.1:0.03, v/v) and injected into an isocratic HPLC consisting of a 10 cm C18 column and UV detector at 254 nm. The plasma corticosterone concentration was measured by an isocratic system using an HPLC pump (model Hewlett Packard Agilent 1100) (flow rate: 1 mL/min; injection volume: 150 μL). Pure corticosterone (Sigma, St. Louis, MO) was provided, dissolved in ethyl acetate. The samples were applied directly and compared with standard pure corticosterone (21).

## Results and Discussion

*Caffeine test in intact and adrenalectomized rats*

While epileptiform activity (tonic-clonic convulsion) was observed 3.40 min after the caffeine injection in intact rats, this time (latent period) was observed 1.51 min after the injection in adrenalectomized rats. All the rats of these two groups died within two hours, but the adrenalectomized rats died before the intact rats (Table 1).

**Table 1:** Comparison of caffeine induced epilepsy degree (epileptiform activity) in intact and adrenalectomized rats

	**Dose (mg/Kg)**	**Number of animals**	**Time of latent period (min.)**	**p**	**Number of convulsive animals**	**Number of dead animals in 2 h**
**Intact rats**						
**Caffeine**	300	8	3.40 ± 0.27	< 0.0001	8	8
**Adrenalectomized rats**						
**Caffeine**	300	8	1.51 ± 0.17	< 0.0001	8	8

*Caffeine test in adrenalectomized rats given adrenaline and prednisolone*

Tonic-clonic convulsion happened 9.04 min after the caffeine injection in the rats that had been administered adrenaline, and in the prednisolone intake group, this period was seen 2.45 min after administration. All of the prednisolone intake rats died within two hours, while none of the rats given adrenaline died (Table 2).

**Table 2:** Effects of adrenaline and prednisolone in caffeine induced epilepsy (epileptiform activity) in adrenalectomized rats

**Drug**	**Dose**	**Number of animals**	**Time of latent period (min.)**	**p**	**Number of convulsive animals**	**Number of dead animals in 2 h**
**Adrenaline**	100 μg/Kg	8	9.04 ± 0.45	< 0.0001	8	-
**Prednisolone**	5 mg/Kg	8	2.45 ± 0.29	< 0.0001	8	8

*Prazosin, phenoxybenzamine, yohimbine, metoprolol, and propranolol tests in adrenalectomized rats*

As seen in Table 3, in the control group receiving adrenaline only, the latent period of caffeine-induced epileptiform activity was 11.0 min, and in the rats given prazosin, phenoxybenzamine, yohimbine, metoprolol, and propranolol, the effect of adrenaline during this period was 7.56, 6.02, 4.03, 4.25, and 2.03, respectively. During this part of the study, the largest number of deaths was observed in the prazosin, phenoxybenzamine, and metoprolol group. The propranolol group had the fewest deaths.

**Table 3:** Effects of adrenaline in prazosin, phenoxybenzamine, yohimbine, metoprolol and propranolol given adrenalectomized rats in caffeine induced epilepsy (epileptiform activity).

**Drug**	**Dose**	**Number of animals**	**Time of latent period (min.)**	**p**	**Number of convulsive animal**	**Number of dead animal in 2 h**
**Prazosin** **+** **Adrenaline**	5 mg/Kg 100 μg/Kg	8	7.56 ± 0.42	< 0.0001	8	4
**Phenoxybenzamine** **+** **Adrenaline**	20 mg/Kg 100 μg/Kg	8	6.02 ± 0.30	< 0.0001	8	4
**Yohimbine** **+** **Adrenaline**	10 mg/Kg 100 μg/Kg	8	4.03 ± 0.23	< 0.0001	8	6
**Metoprolol** **+** **Adrenaline**	50 mg/Kg 100 μg/Kg	8	4.25 ± 0.28	< 0.0001	8	5
**Propranolol** **+** **Adrenaline**	40 mg/Kg 100 μg/Kg	8	2.03 ± 0.21	< 0.0001	8	8
**Adrenaline (Control)**	100 μg/Kg	8	11.00 ± 0.30	-	8	-

*Acute indomethacin test in intact rats*

In the 10 mg/Kg single-dose indomethacin group, tonic-clonic convulsions appeared 3.44 min after the caffeine injection, and in the control group, this time was 3.06 min. All of the rats died within two hours (Table 4).

**Table 4:** Effect of acute (single dose) administration of indomethacin on caffeine induced epilepsy (epileptiform activity) in rats

**Drug**	**Dose (mg/Kg)**	**Number of animals**	**Time of latent period (min.)**	**p**	**Number of convulsive animal**	**Number of dead animal in 2 h**
**Intact rats**						
**Indomethacin**	10	8	3.44 ± 0.28	> 0.05	8	8
**Control (Caffeine)**	300	8	3.06 ± 0.22	> 0.05	8	8
**Adrenalectomized rats**						
**Indomethacin**	10	8	1.37 ± 0.15	> 0.05	8	8
**Control (Caffeine)**	300	8	1.18 ± 0.06	> 0.05	8	8

*Acute indomethacin test in adrenalectomized rats*

In the 10 mg/Kg single-dose indomethacin group, the latent period was 3.44 min, and in the control group, this time was 3.06 min. All of the rats died within 2 h (Table 4).

*Chronic indomethacin test in intact rats*

In the chronic-dose indomethacin group, the tonic-clonic convulsion latent period was 2.03 min, and in the control group, this time was extended to 3.06 min. All of the rats died within two hours (Table 5).

**Table 5:** Effect of chronicle administration of indomethacin on caffeine induced epilepsy (epileptiform activity) in rats

**Drug**	**Dose (mg/Kg)**	**Number of animals**	**Time of latent period (min.)**	**p**	**Number of convulsive animal**	**Number of dead animal in 2 h**
**Intact rats**						
**Indomethacin**	10	8	2.03 ± 0.22	< 0.0001	8	8
**Control** **(Caffeine)**	300	8	4.19 ± 0.30	< 0.0001	8	8
**Adrenalectomized rats**						
**Indomethacin**	10	8	2.21 ± 0.23	> 0.05	8	8
**Control** **(Caffeine)**	300	8	2.30 ± 0.17	> 0.05	8	8

*Chronic indomethacin test in adrenalectomized rats*

In the chronic indomethacin administered group, the latent period was 2.21 min, and in the control group, this time was 2.30 min. All of the rats died within two hours (Table 5).

*Effect of acute and chronic indomethacin intake on blood adrenaline, noradrenaline, dopamine, and corticosterone levels in rats*

**Figure 1:**
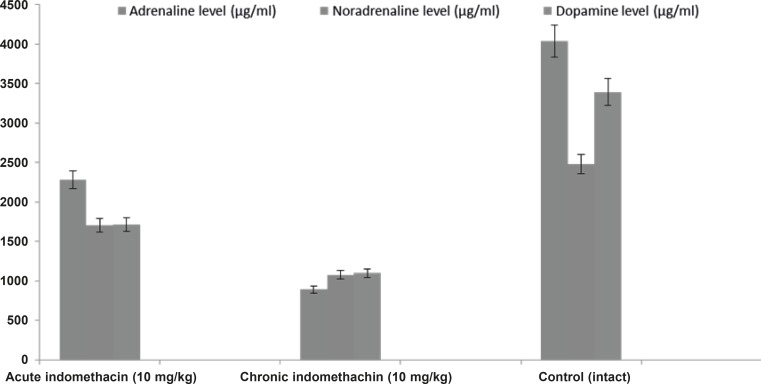
Effects of acute and chronic indomethacin administration on adrenalin, noradrenaline and dopamine levels in rats. n = 8; * refers p < 0.05

**Figure 2:**
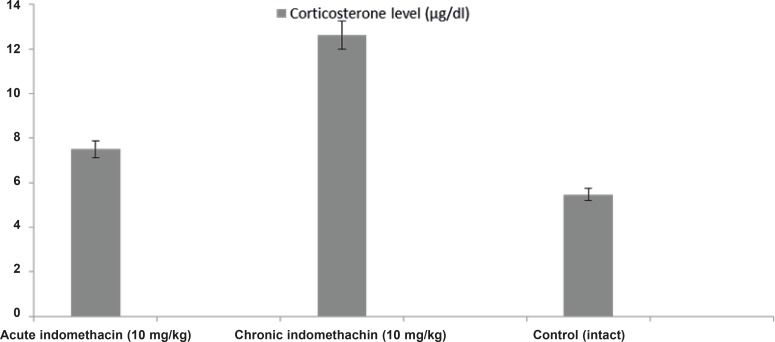
Effects of acute and chronic indomethacin administration on corticosterone levels in rats. n = 8; * refers p < 0.05

In the 10 mg/Kg single-dose indomethacin intake rats, the adrenaline, noradrenaline, dopamine, and corticosterone levels were 2280.0 ± 280.8 μg/mL, 1706.3 ± 149.4 μg/mL, 1710.5 ± 173.9 μg/mL, and 7.5 ± 0.32 μg/dL, respectively, and in the chronic group, which was given indomethacin over 7 days, these levels were 889.9 ± 130.4 μg/mL, 1077.6 ± 63.5 μg/mL, 1099.0 ± 38.9 μg/mL, and 12.63 ± 0.39 μg/dL, respectively. In the intact rats, the levels were 4042.2 ± 426.7 μg/mL, 2481.4 ± 140.9 μg/mL, 3393.6 ± 118.1 μg/mL, and 5.46 ± 0.23 μg/dL, respectively (Figures 1 and 2).

In this study, the effects of adrenaline, prednisolone, and indomethacin on caffeine-induced epileptiform activity were investigated in rats. In addition, the mechanism of the pro-epileptic effect of indomethacin was examined.

To investigate the antiepileptic activity of adrenaline, first the degree of epileptiform activity induced by caffeine was compared in adrenalectomized and intact rats. The results showed that tonic-clonic convulsions started in adrenalectomized rats before starting in the intact rats. The difference in latent periods between the adrenalectomized and intact rats was statistically significant. It is well known that adrenaline is not synthesized in adrenergic synapses (11), and corticosterones in low doses do not change the degree of epilepsy (13). Furthermore, an increase in corticosterone level or chronic corticosteroid treatment elevates the epileptogenesis (13, 22). According to our results and knowledge of the literature, it can be said that the decrease in adrenaline level is responsible for the decrease in the latent period in the group that underwent adrenalectomy.

On the other hand, to decide whether adrenaline and cortisol (corticosterone in rats) have an anti-epileptic effect exactly, we investigated the effects of these drugs on epileptiform activity in adrenalectomized rats. The results showed that in adrenalectomized rats given adrenaline, the latent period is 3.7 times longer than that of the cortisol group (prednisolone was used instead of cortisol). As mentioned above, we did not find any reports in the literature regarding the anti-epileptic activity of adrenaline. But in the literature it was seen that adrenaline receptors are widely found in the central nerve system (23, 24). In addition, it has been reported that adrenergic receptors were responsible for antiepileptic activity (15, 25). It was also reported that the preventive effect of noradrenaline on pentylenetetrazol-induced epilepsy was conducted via alpha-1 adrenergic receptors (26).

In this study, we found that in the adrenalectomized rats given prazosin + adrenaline, the latent period shortened significantly versus the control rat group that was given only adrenaline. Moreover, in the adrenalectomized rats that were given phenoxybenzamine, yohimbine, metoprolol, and propranolol before the adrenaline injection, the latent period shortened significantly versus the control group given adrenaline. The adrenalectomized rats that had been given only adrenaline (the control group) did not die; however, adrenaline did not prevent the adrenalectomized rats given prazosin, phenoxybenzamine, yohimbine, metoprolol, and propranolol from dying. In the rats given propranolol + adrenaline, the rate of death was 100%, while this rate was 50% in the groups given prazosin + adrenaline, phenoxybenzamine + adrenaline, and metoprolol + adrenaline. The rate was 75% in the group given yohimbine + adrenaline. These results have told us that in antiepileptic activity *β*-2 adrenergic receptors play a more important role than the other adrenergic receptors. It has been understood that *α*-1 and *β*-1 receptors are the least important receptors in antiepileptic activity.

It was demonstrated that *α*-2 adrenergic receptors had subtypes such as *α*-2A, *α*-2B, and *α*-2C in the central nerve system (27). It was reported that noradrenaline decreased epileptiform activity via activating *α*-2A receptors in the hippocampal region; selective *α*-2adrenergic receptor antagonists (yohimbine) inhibited this antiepileptic activity as well (28). Another study revealed that *β*-1adrenergic receptors had no role in preventing pentylenetetrazol seizures (26). This knowledge partly supported our results.

This investigation demonstrated that prednisolone increased the degree of convulsion in adrenalectomized rats. In epilepsy patients, the number of glucocorticoid (GR) and mineralocorticoid receptors (MR) is enhanced (29). Similarly, in adrenalectomized rats, the number of these receptors increased (30). For this reason, glucocorticoid (prednisolone) treatment increased the degree of epileptiform activity in adrenalectomized rats.

Interaction of caffeine with GABA_A_/benzodiazepine receptors has explained the various central effects and the high-dose convulsing effect of caffeine (31). Adrenaline prevented caffeine-induced convulsions, and this anticonvulsant effect (epileptiform activity) was antagonized by adrenergic receptor blockers.

It was found that adrenaline had a potent anti-inflammatory effect, and this effect was antagonized by *β*-2 adrenergic receptor blocker (14). In addition, it was shown that adrenaline composed a gastro-protective effect via *α*-2 adrenergic receptors (18). This means that adrenergic receptor agonists can have antiepileptic activity. All of the drugs and substances that block adrenergic receptors can trigger epilepsy genesis. N. Maisov *et al*. showed that in an oxygen-induced epilepsy model, adrenaline, dopamine, and noradrenaline metabolites disappear in the brain 5 min after oxygen is given (32).

Studies have pointed out drugs that decrease adrenaline, noradrenaline, and dopamine levels and increase the cortisol (corticosterone in rats) level can elevate the degree and frequency of epilepsy. In light of these findings, the effect of indomethacin, contraindicated in epilepsy, on epileptiform activity and the blood levels of adrenaline, noradrenaline, dopamine, and corticosterone was investigated. In the single (acute) dose indomethacin rat group, the latent period of epileptiform activity increased, but this longer duration was statistically insignificant. However, chronic indomethacin administration (7 days) caused a significant decrease in the latent period. The increase in epileptiform activity by chronic indomethacin administration supported the clinical contraindication of this drug.

Biochemical analysis demonstrated that catecholamines (adrenaline, noradrenaline, and dopamine) in the rats given single-dose indomethacin decreased significantly versus the control group. In the chronic (7 days) indomethacin group, the catecholamine levels measured as lower than those of the acute group. In addition, in the rats given chronic indomethacin, the corticosterone levels made a significant peak versus the single-dose group. As mentioned above, low-dose corticosterone did not change the degree of epilepsy, but high doses increased the degree of epilepsy (13). The increase in the corticosterone level augmented the epileptogenesis degree by changing the hippocampal cell functions (22, 33). Corticosteroids caused stimulation of these cells by increasing Ca^++^ entrance to CA1 cells (34).

Chronic corticosterone secretion increased the limbic epileptogenesis; GR and MR blockers (spironolactone, mifepristone) inhibited this increase (35).

In conclusion, over-reduction in the blood catecholamine level made epileptogenesis more severe. It was observed that adrenaline pressed epileptogenesis via its own receptors (*α*-1, *α*-2, *β*-1, *β*-2). It was revealed that all of the adrenergic receptors were responsible due to antiepileptic activity; *β*-2 receptors played the most important role. It was also observed that both acute and chronic indomethacin administration reduced the catecholamine levels. The situation in which acute administration of indomethacin did not affect epileptogenesis might be because the structure of indomethacin did not significantly increase the corticosterone level. Epileptogenesis increasing the effect of chronic indomethacin administration might come from the clear corticosterone-increasing action. 
